# Diphenyl[2-(phenyl­sulfon­yl)propan-2-yl]-λ^5^-phosphane­thione

**DOI:** 10.1107/S1600536812010082

**Published:** 2012-03-14

**Authors:** Viktoria H. Gessner

**Affiliations:** a Institut für Anorganische Chemie, Julius-Maximilians-Universität Würzburg, Am Hubland, 97074 Würzburg, Germany

## Abstract

The title compound, C_21_H_21_O_2_PS_2_, was obtained from the corresponding dilithio methandiide by treatment with iodo­methane. The bond lengths and angles deviate considerably from those in the dimetallated compound. These differences are most pronounced in the PCS backbone. While the title compound features C—P and C—S distances of 1.9082 (17) and 1.8348 (17) Å, respectively, the dianion showed C—P_av_ distances shortened by 11% [1.710 (4) Å] and C—S distances shortened by 12% [1.614 (3) Å]. Additionally, the P—C—S angle experiences a contraction by methyl­ation of the dianion from 121.4 (2) to 111.96 (9)° in the title compound.

## Related literature

For background to precursors for dilithio methandiides, see: Kasani *et al.* (1999 ▶); Ong & Stephan (1999 ▶); Cantat *et al.* (2006 ▶, 2008 ▶); Cavell *et al.* (2001 ▶); Harder (2011 ▶); Gessner (2011 ▶); Gessner & Schröter (2012 ▶); Cooper *et al.* (2010 ▶).
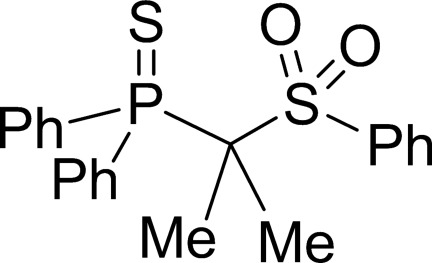


## Experimental

### 

#### Crystal data

C_21_H_21_O_2_PS_2_
*M* *_r_* = 400.47
Orthorhombic, 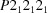

*a* = 8.2137 (7) Å
*b* = 14.3714 (13) Å
*c* = 16.6728 (15) Å
*V* = 1968.1 (3) Å^3^
*Z* = 4
Mo *K*α radiation
μ = 0.37 mm^−1^
*T* = 173 K
0.26 × 0.2 × 0.16 mm

#### Data collection

Bruker APEX CCD diffractometer
Absorption correction: multi-scan (*SADABS*; Bruker, 1999 ▶) *T*_min_ = 0.973, *T*_max_ = 0.980
30953 measured reflections
3457 independent reflections
3373 reflections with *I* > 2σ(*I*)
*R*_int_ = 0.040

#### Refinement

*R*[*F* ^2^ > 2σ(*F* ^2^)] = 0.025
*wR*(*F* ^2^) = 0.068
*S* = 1.04
3457 reflections
237 parameters
H-atom parameters constrained
Δρ_max_ = 0.27 e Å^−3^
Δρ_min_ = −0.13 e Å^−3^
Absolute structure: Flack (1983 ▶), 1467 Friedel pairs
Flack parameter: 0.03 (5)

### 

Data collection: *SMART* (Bruker, 2001 ▶); cell refinement: *SAINT-Plus* (Bruker, 1999 ▶); data reduction: *SAINT-Plus*; program(s) used to solve structure: *SHELXS97* (Sheldrick, 2008 ▶); program(s) used to refine structure: *SHELXL97* (Sheldrick, 2008 ▶); molecular graphics: *ORTEP-3* (Farrugia, 1997 ▶); software used to prepare material for publication: *SHELXL97*.

## Supplementary Material

Crystal structure: contains datablock(s) I, global. DOI: 10.1107/S1600536812010082/bt5837sup1.cif

Structure factors: contains datablock(s) I. DOI: 10.1107/S1600536812010082/bt5837Isup2.hkl

Supplementary material file. DOI: 10.1107/S1600536812010082/bt5837Isup3.cml

Additional supplementary materials:  crystallographic information; 3D view; checkCIF report

## Figures and Tables

**Table 1:** Selected bond lengths (Å)

S2—O2	1.4347 (13)
S2—O1	1.4389 (13)
S2—C16	1.7734 (16)
S2—C13	1.8348 (17)
P1—C1	1.8178 (17)
P1—C7	1.8263 (17)
P1—C13	1.9082 (17)
P1—S1	1.9515 (6)

**Fig. 1.**
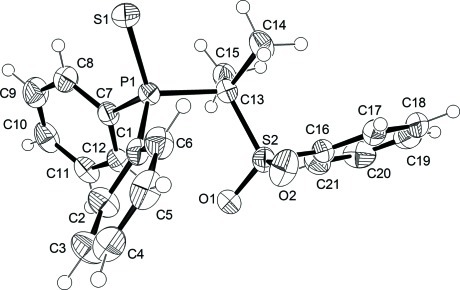
The molecular structure of the title compound. Displacement ellipsoids are drawn at the 50% probability level.

## supplementary crystallographic information

### Comment

Methylene compounds with two anion stabilizing substituents, such as phosphonium or sulfonyl moieties, have become interesting reagents due to their possible transformation into the corresponding methandiide by double deprotonation. (Kasani *et al.*, 1999; Ong *et al.*, 1999; Cantat *et al.*, 2006) These geminal dianions have caught special attention because of their unique electronic properties and structures, which enabled the isolation of a planar four-coordinate carbon atom (Cooper *et al.*, 2010). Additionally, these dianionic species have proven to be efficient ligand systems for the construction of novel carbene complexes, which differ from the known Fischer and Schrock-type complexes. Thereby, a huge variety of different compounds incorporating early and late transition metals as well as lanthanoids and actinoids have been reported (Cavell *et al.*, 2001; Cantat *et al.*, 2008; Harder, 2011).

As part of our studies on dilithio methandiides we developed a new methylene coumpound with both, a thiophosphinoyl and a sulfonyl moiety (Gessner, 2011; Gessner *et al.*, 2012). This compound is easily converted into its dianionic congener, which features strongly distorted geometries of the metallated carbon atoms, which can be explained by a novel bonding mode of a *sp*^2^-hybridized carbon with the two lithium atoms. Treatment of the dilithio methandiide with iodomethane furnished the dimethylated titel compound. The bond lengths and angles are comparable to the protonated analogue, but deviate considerably from the dimetallated compound. These differences are most pronounced in the PCS backbone. While the title compound features C—P and C—S distances of 1.908 (2) and 1.835 (2) Å, respectively, the dianion showed C—P_av_ distances shortened by 11% [1.710 (4) Å] and C—S distances shortened by 12% [1.614 (3) Å] (Gessner *et al.*, 2012). Additionally, the P—C—S angle experiences a contraction by methylation of the dianion from 121.4 (2)° to 112.0 (1)° in the title compound. This is the result of the re-hybridization from a *sp*^2^-hybridized carbon in the methandiide to an *sp*^3^-hybridized carbon in the title compound.

### Experimental

284 mg (2.00 mmol) iodomethane were added to a suspension of 405 mg (0.20 mmol, equates to 0.81 mmol per molecule) of the corresponding dilithio methandiide in 20 ml THF at room temperature. After stirring for 4 h the mixture was treated with 20 ml of an aqueous ammonia solution (25%) and the mixture extracted with diethyl ether. Drying over sodium sulfate and removal of the solvent *in vacuo* afforded the crude product as orange oil. The product was obtained as colorless solid after purification by flash chromatography (eluent: pentane/diethyl ether= 1:1, *R*_F_ = 0.33) on silica (285 mg, 0.71 mmol; 88%). Single crystals were grown by slow evaporation of a solution of the title compound in THF. ^1^H NMR (500.1 MHz, CDCl_3_): δ = 1.63 (d, ^2^*J*_HP_ = 15.6 Hz, 2H; PCC*H_3_*), 7.47–7.58 (m, 6H; C*H*_PPh_ + C*H*_SPh_), 7.62 (tt, ^3^*J*_HH_ = 7.49 Hz, ^4^*J*_HH_ = 1.24 Hz, 1H; C*H*_SPh,_*_para_*),7.77 (dd, ^3^*J*_HH_ =8.46 Hz, ^4^*J*_HH_ =1.22 Hz, 2H; C*H*_SPh,_*_ortho_*),8.36–8.40 (m, 4H; C*H*_Ph,_*_ortho_*).^13^C NMR (125.8 MHz, CDCl_3_): δ = 22.8 [d, ^2^*J*_CP_ = 2.2 Hz; PC(*C*H_3_)_2_], 67.8 [d, ^1^*J*_CP_ = 34.1 Hz; P*C*(CH_3_)_2_], 128.0(d, ^2^*J*_CP_ = 12.8 Hz; *C*H*_ortho_*), 128.7 (*C*H_SPh_), 129.3 (d, ^1^*J*_CP_ = 80.4 Hz; *C_ipso_*), 130.3 (*C*H_SPh_), 132.0 (d, ^4^*J*_CP_ = 3.2 Hz; *C*H*_para_*), 133.9 (*C*H_SPh,_*_para_*), 134.2 (d, ^3^*J*_CP_ = 10.3 Hz; *C*H*_meta_*), 140.1 (d, ^3^*J*_CP_ = 0.8 Hz; S*C*_Ph_). ^31^P{H} NMR (162.0 MHz, CDCl_3_):δ = 58.7. Anal. Calcd for C_19_H_17_PO_2_S_2_:*C*, 62.98; H, 5.29; S 16.01. Found: C, 63.14; H, 5.31; S 16.41.

### Refinement

H atoms were located in a difference map and refined as riding with C-H ranging from 0.95Å to 0.98Å and U(H) set to 1.2U_eq_(C) or 1.5U_eq_(C_methyl_).

### Crystal data

**Table:**

C_21_H_21_O_2_PS_2_	*D*_x_ = 1.352 Mg m^−^^3^
*M**_r_* = 400.47	Melting point: 410 K
Orthorhombic, *P*2_1_2_1_2_1_	Mo *K*α radiation, λ = 0.71073 Å
Hall symbol: P 2ac 2ab	Cell parameters from 3457 reflections
*a* = 8.2137 (7) Å	θ = 1.9–25°
*b* = 14.3714 (13) Å	µ = 0.37 mm^−^^1^
*c* = 16.6728 (15) Å	*T* = 173 K
*V* = 1968.1 (3) Å^3^	Block, colourless
*Z* = 4	0.26 × 0.2 × 0.16 mm
*F*(000) = 840	

### Data collection

**Table:**

Bruker APEX CCD diffractometer	3457 independent reflections
Radiation source: fine-focus sealed tube	3373 reflections with *I* > 2σ(*I*)
Graphite monochromator	*R*_int_ = 0.040
ω scans	θ_max_ = 25°, θ_min_ = 1.9°
Absorption correction: multi-scan (*SADABS*; Bruker, 1999)	*h* = −9→9
*T*_min_ = 0.973, *T*_max_ = 0.980	*k* = −17→17
30953 measured reflections	*l* = −19→19

### Refinement

**Table:**

Refinement on *F*^2^	Secondary atom site location: difference Fourier map
Least-squares matrix: full	Hydrogen site location: inferred from neighbouring sites
*R*[*F*^2^ > 2σ(*F*^2^)] = 0.025	H-atom parameters constrained
*wR*(*F*^2^) = 0.068	*w* = 1/[σ^2^(*F*_o_^2^) + (0.0456*P*)^2^ + 0.2311*P*] where *P* = (*F*_o_^2^ + 2*F*_c_^2^)/3
*S* = 1.04	(Δ/σ)_max_ = 0.006
3457 reflections	Δρ_max_ = 0.27 e Å^−^^3^
237 parameters	Δρ_min_ = −0.13 e Å^−^^3^
0 restraints	Absolute structure: Flack (1983), 1467 Friedel pairs
Primary atom site location: structure-invariant direct methods	Flack parameter: 0.03 (5)

### Special details

**Table:**

Geometry. All e.s.d.'s (except the e.s.d. in the dihedral angle between two l.s. planes) are estimated using the full covariance matrix. The cell e.s.d.'s are taken into account individually in the estimation of e.s.d.'s in distances, angles and torsion angles; correlations between e.s.d.'s in cell parameters are only used when they are defined by crystal symmetry. An approximate (isotropic) treatment of cell e.s.d.'s is used for estimating e.s.d.'s involving l.s. planes.
Refinement. Refinement of *F*^2^ against ALL reflections. The weighted *R*-factor *wR* and goodness of fit *S* are based on *F*^2^, conventional *R*-factors *R* are based on *F*, with *F* set to zero for negative *F*^2^. The threshold expression of *F*^2^ > σ(*F*^2^) is used only for calculating *R*-factors(gt) *etc*. and is not relevant to the choice of reflections for refinement. *R*-factors based on *F*^2^ are statistically about twice as large as those based on *F*, and *R*- factors based on ALL data will be even larger.

### Fractional atomic coordinates and isotropic or equivalent isotropic displacement parameters (Å^2^)

**Table:**

	*x*	*y*	*z*	*U*_iso_*/*U*_eq_	
S2	0.17343 (5)	0.02033 (3)	0.10827 (2)	0.03362 (11)	
P1	0.00880 (5)	−0.07757 (3)	0.25295 (3)	0.03106 (11)	
S1	0.08167 (6)	−0.16347 (4)	0.33619 (3)	0.04775 (14)	
O1	0.01826 (15)	0.06395 (9)	0.09446 (8)	0.0433 (3)	
C12	−0.2075 (2)	0.07773 (11)	0.24561 (11)	0.0352 (4)	
H12	−0.1707	0.0882	0.1923	0.042*	
O2	0.21542 (19)	−0.06103 (9)	0.06291 (8)	0.0501 (4)	
C13	0.1913 (2)	−0.00806 (11)	0.21520 (10)	0.0337 (4)	
C17	0.4808 (2)	0.07625 (13)	0.06696 (11)	0.0396 (4)	
H17	0.5025	0.0122	0.0577	0.048*	
C21	0.2932 (2)	0.19798 (12)	0.10399 (13)	0.0441 (4)	
H21	0.1879	0.2172	0.1207	0.053*	
C16	0.3273 (2)	0.10474 (11)	0.09172 (10)	0.0334 (4)	
C6	−0.0079 (2)	−0.21832 (12)	0.14063 (12)	0.0428 (4)	
H6	0.0979	−0.234	0.1594	0.051*	
C2	−0.2416 (2)	−0.12018 (13)	0.14386 (12)	0.0429 (4)	
H2	−0.2971	−0.0672	0.1643	0.051*	
C7	−0.14183 (19)	0.00496 (11)	0.29077 (10)	0.0326 (4)	
C20	0.4151 (3)	0.26272 (14)	0.09151 (14)	0.0558 (5)	
H20	0.393	0.327	0.0991	0.067*	
C11	−0.3259 (2)	0.13455 (13)	0.27833 (12)	0.0426 (4)	
H11	−0.3702	0.1839	0.2473	0.051*	
C9	−0.3160 (3)	0.04939 (16)	0.40124 (13)	0.0542 (5)	
H9	−0.3534	0.0396	0.4545	0.065*	
C18	0.6012 (2)	0.14149 (15)	0.05597 (12)	0.0487 (5)	
H18	0.7071	0.1224	0.04	0.058*	
C3	−0.3162 (3)	−0.17589 (13)	0.08735 (13)	0.0510 (5)	
H3	−0.4232	−0.1615	0.0696	0.061*	
C4	−0.2356 (3)	−0.25289 (13)	0.05628 (12)	0.0463 (5)	
H4	−0.2864	−0.2909	0.017	0.056*	
C15	0.2017 (2)	0.08108 (14)	0.26490 (11)	0.0428 (4)	
H15A	0.1919	0.0657	0.322	0.064*	
H15B	0.1132	0.1232	0.2494	0.064*	
H15C	0.3066	0.1115	0.2552	0.064*	
C10	−0.3803 (2)	0.12028 (14)	0.35551 (13)	0.0499 (5)	
H10	−0.4624	0.1594	0.3773	0.06*	
C8	−0.1966 (2)	−0.00755 (14)	0.36918 (12)	0.0452 (4)	
H8	−0.1514	−0.0558	0.4011	0.054*	
C5	−0.0821 (3)	−0.27321 (12)	0.08296 (12)	0.0463 (5)	
H5	−0.0259	−0.3255	0.0617	0.056*	
C14	0.3465 (2)	−0.06516 (15)	0.22535 (14)	0.0509 (5)	
H14A	0.4401	−0.0288	0.2067	0.076*	
H14B	0.3381	−0.1225	0.1938	0.076*	
H14C	0.3611	−0.0809	0.2821	0.076*	
C1	−0.0864 (2)	−0.14097 (11)	0.17107 (10)	0.0317 (3)	
C19	0.5685 (3)	0.23456 (15)	0.06813 (12)	0.0523 (5)	
H19	0.652	0.2794	0.0604	0.063*	

### Atomic displacement parameters (Å^2^)

**Table:**

	*U*^11^	*U*^22^	*U*^33^	*U*^12^	*U*^13^	*U*^23^
S2	0.0321 (2)	0.0363 (2)	0.0325 (2)	−0.00442 (18)	0.00327 (17)	−0.00215 (17)
P1	0.0248 (2)	0.0357 (2)	0.0327 (2)	0.00245 (16)	0.00071 (17)	0.00384 (16)
S1	0.0398 (2)	0.0565 (3)	0.0469 (3)	0.0057 (2)	−0.0028 (2)	0.0196 (2)
O1	0.0308 (6)	0.0597 (8)	0.0395 (7)	−0.0048 (6)	−0.0033 (5)	0.0047 (6)
C12	0.0282 (8)	0.0387 (8)	0.0388 (9)	−0.0012 (7)	−0.0004 (7)	−0.0033 (8)
O2	0.0647 (9)	0.0389 (7)	0.0468 (7)	−0.0110 (6)	0.0159 (7)	−0.0096 (6)
C13	0.0262 (8)	0.0406 (9)	0.0343 (8)	−0.0002 (7)	0.0015 (7)	0.0020 (7)
C17	0.0384 (10)	0.0442 (9)	0.0362 (9)	0.0016 (8)	0.0051 (8)	0.0022 (8)
C21	0.0409 (10)	0.0395 (9)	0.0520 (11)	0.0027 (8)	−0.0034 (9)	0.0023 (8)
C16	0.0301 (8)	0.0363 (8)	0.0336 (9)	−0.0033 (7)	−0.0003 (7)	0.0019 (7)
C6	0.0393 (10)	0.0383 (9)	0.0507 (11)	0.0063 (8)	0.0059 (9)	0.0019 (8)
C2	0.0367 (9)	0.0391 (9)	0.0528 (11)	0.0073 (8)	−0.0037 (9)	−0.0119 (8)
C7	0.0262 (8)	0.0381 (9)	0.0334 (9)	−0.0019 (6)	0.0014 (6)	−0.0044 (7)
C20	0.0681 (14)	0.0369 (9)	0.0625 (14)	−0.0084 (10)	−0.0144 (12)	0.0073 (9)
C11	0.0316 (9)	0.0410 (9)	0.0551 (11)	0.0013 (8)	−0.0035 (8)	−0.0105 (8)
C9	0.0466 (11)	0.0731 (13)	0.0428 (11)	−0.0010 (10)	0.0133 (10)	−0.0128 (10)
C18	0.0327 (10)	0.0758 (13)	0.0376 (10)	−0.0093 (10)	0.0010 (8)	0.0072 (9)
C3	0.0427 (10)	0.0493 (10)	0.0610 (12)	−0.0003 (10)	−0.0104 (10)	−0.0145 (10)
C4	0.0602 (13)	0.0377 (9)	0.0410 (10)	−0.0101 (9)	0.0042 (9)	−0.0057 (8)
C15	0.0343 (9)	0.0573 (11)	0.0369 (9)	−0.0097 (8)	−0.0008 (7)	−0.0075 (8)
C10	0.0339 (10)	0.0558 (11)	0.0599 (13)	0.0003 (9)	0.0081 (9)	−0.0244 (10)
C8	0.0415 (10)	0.0577 (11)	0.0366 (9)	−0.0013 (9)	0.0046 (8)	0.0010 (8)
C5	0.0596 (12)	0.0297 (8)	0.0495 (11)	0.0037 (9)	0.0166 (10)	−0.0038 (8)
C14	0.0277 (9)	0.0625 (12)	0.0623 (12)	0.0066 (9)	0.0042 (9)	0.0142 (10)
C1	0.0296 (8)	0.0291 (7)	0.0363 (9)	−0.0011 (7)	0.0050 (7)	0.0027 (6)
C19	0.0512 (12)	0.0626 (13)	0.0431 (11)	−0.0239 (11)	−0.0102 (10)	0.0102 (9)

### Geometric parameters (Å, º)

**Table:**

S2—O2	1.4347 (13)	C7—C8	1.394 (3)
S2—O1	1.4389 (13)	C20—C19	1.380 (3)
S2—C16	1.7734 (16)	C20—H20	0.9500
S2—C13	1.8348 (17)	C11—C10	1.378 (3)
P1—C1	1.8178 (17)	C11—H11	0.9500
P1—C7	1.8263 (17)	C9—C10	1.378 (3)
P1—C13	1.9082 (17)	C9—C8	1.384 (3)
P1—S1	1.9515 (6)	C9—H9	0.9500
C12—C11	1.382 (2)	C18—C19	1.379 (3)
C12—C7	1.397 (2)	C18—H18	0.9500
C12—H12	0.9500	C3—C4	1.390 (3)
C13—C14	1.526 (2)	C3—H3	0.9500
C13—C15	1.528 (2)	C4—C5	1.368 (3)
C17—C18	1.375 (3)	C4—H4	0.9500
C17—C16	1.388 (2)	C15—H15A	0.9800
C17—H17	0.9500	C15—H15B	0.9800
C21—C20	1.382 (3)	C15—H15C	0.9800
C21—C16	1.384 (2)	C10—H10	0.9500
C21—H21	0.9500	C8—H8	0.9500
C6—C1	1.382 (2)	C5—H5	0.9500
C6—C5	1.385 (3)	C14—H14A	0.9800
C6—H6	0.9500	C14—H14B	0.9800
C2—C3	1.380 (3)	C14—H14C	0.9800
C2—C1	1.386 (3)	C19—H19	0.9500
C2—H2	0.9500		
			
O2—S2—O1	118.92 (9)	C10—C11—C12	120.60 (18)
O2—S2—C16	107.71 (8)	C10—C11—H11	119.7
O1—S2—C16	107.96 (8)	C12—C11—H11	119.7
O2—S2—C13	108.16 (8)	C10—C9—C8	119.67 (19)
O1—S2—C13	108.87 (7)	C10—C9—H9	120.2
C16—S2—C13	104.26 (8)	C8—C9—H9	120.2
C1—P1—C7	107.07 (8)	C17—C18—C19	120.1 (2)
C1—P1—C13	110.64 (8)	C17—C18—H18	119.9
C7—P1—C13	107.83 (8)	C19—C18—H18	119.9
C1—P1—S1	110.42 (6)	C2—C3—C4	120.36 (19)
C7—P1—S1	111.90 (6)	C2—C3—H3	119.8
C13—P1—S1	108.95 (6)	C4—C3—H3	119.8
C11—C12—C7	120.08 (17)	C5—C4—C3	119.18 (19)
C11—C12—H12	120.0	C5—C4—H4	120.4
C7—C12—H12	120.0	C3—C4—H4	120.4
C14—C13—C15	110.14 (16)	C13—C15—H15A	109.5
C14—C13—S2	107.12 (13)	C13—C15—H15B	109.5
C15—C13—S2	110.18 (12)	H15A—C15—H15B	109.5
C14—C13—P1	109.78 (12)	C13—C15—H15C	109.5
C15—C13—P1	107.68 (11)	H15A—C15—H15C	109.5
S2—C13—P1	111.96 (9)	H15B—C15—H15C	109.5
C18—C17—C16	119.45 (17)	C9—C10—C11	120.16 (18)
C18—C17—H17	120.3	C9—C10—H10	119.9
C16—C17—H17	120.3	C11—C10—H10	119.9
C20—C21—C16	118.89 (19)	C9—C8—C7	120.96 (19)
C20—C21—H21	120.6	C9—C8—H8	119.5
C16—C21—H21	120.6	C7—C8—H8	119.5
C21—C16—C17	120.86 (17)	C4—C5—C6	120.64 (18)
C21—C16—S2	119.68 (14)	C4—C5—H5	119.7
C17—C16—S2	119.46 (13)	C6—C5—H5	119.7
C1—C6—C5	120.52 (18)	C13—C14—H14A	109.5
C1—C6—H6	119.7	C13—C14—H14B	109.5
C5—C6—H6	119.7	H14A—C14—H14B	109.5
C3—C2—C1	120.44 (17)	C13—C14—H14C	109.5
C3—C2—H2	119.8	H14A—C14—H14C	109.5
C1—C2—H2	119.8	H14B—C14—H14C	109.5
C8—C7—C12	118.51 (16)	C6—C1—C2	118.84 (17)
C8—C7—P1	117.30 (14)	C6—C1—P1	118.59 (14)
C12—C7—P1	124.17 (13)	C2—C1—P1	122.23 (13)
C19—C20—C21	120.42 (19)	C20—C19—C18	120.23 (19)
C19—C20—H20	119.8	C20—C19—H19	119.9
C21—C20—H20	119.8	C18—C19—H19	119.9
